# Low Cardiovascular Disease Awareness in Chilean Women: Insights from the ESCI Project

**DOI:** 10.5334/gh.534

**Published:** 2020-08-12

**Authors:** Paola Varleta, Mónica Acevedo, Carolina Casas-Cordero, Amalia Berríos, Carlos Navarrete

**Affiliations:** 1 Fundación SOCHICAR, Sociedad Chilena de Cardiología y Cirugía Cardiovascular, Santiago, CL; 2 Centro Cardiovascular, Hospital DIPRECA, Santiago, CL; 3 División de Enfermedades Cardiovasculares, Escuela de Medicina, Facultad de Medicina, Pontificia Universidad Católica de Chile, Santiago, CL; 4 Instituto de Ciencias Sociales, Pontificia Universidad Católica de Chile, Santiago, CL; 5 Centro de Encuestas y Estudios Longitudinales, Pontificia Universidad Católica de Chile, Santiago, CL; 6 Facultad de Matemáticas, Universidad de la Serena, La Serena, CL

**Keywords:** cardiovascular disease, women, risk factors, health knowledge

## Abstract

**Background:**

Although cardiovascular disease (CVD) is the leading cause of mortality in Latin American women, limited data exist on CVD perceptions in this population. This study aimed to assess CVD awareness and knowledge of women from Santiago, Chile.

**Methods:**

This was a cross-sectional study conducted in women 35 to 70 years old. A multistage probability sampling (stratified by age and socioeconomic level) was used for participant selection. Participants completed a home survey about knowledge of CVD, risk factors, and perceived risk (based on standardized questions from the American Heart Association awareness survey).

**Results:**

723 women participated in the study (mean age: 51 ± 9 years; 17.6% with high education level). Only 9.3% of the respondents mentioned CVD as women’s primary health problem, whereas 22.7% and 16.1%, respectively, listed breast cancer and other cancers. When asked to identify the leading cause of women’s death, only 14.4% identified CVD compared to 69.1% who recorded cancer. Older women (≥ 55 years) more likely identified CVD as the main cause of death: (OR 2.9: 95% CI = 1.8–4.5) versus younger women (<55 years). CVD family history was also associated with higher awareness of CVD as the leading cause of death (OR 1.7: 95% IC; p = 1.1–2.6). Instead, women with middle education level were less likely to mention CVD as the main women’s killer.

**Conclusions:**

Chilean women from Santiago have a low awareness of CVD as the leading cause of death and do not recognize CVD as their prominent health problem. Efforts should focus on increasing awareness and knowledge about CVD especially in young women.

## Introduction

Cardiovascular disease (CVD), including coronary heart disease (CHD) and stroke, is the leading cause of death in women from developed and developing countries. Each year, more than 8.5 million women die of CVD worldwide, representing approximately one third of all deaths among women [1,2]. Compared with men, younger women who have had an acute coronary syndrome have higher mortality than men of the same age [3]. However, women tend to underestimate the threat CVD poses to them and generally consider it to be a health problem for men.

This poor CVD awareness in women was first reported in 1997 by Mosca and colleagues. They conducted a survey commissioned by the American Heart Association (AHA) to assess awareness and knowledge of CVD and stroke, their risk factors, and preventive behaviors in women in the United States. The study showed that less than a third of the women who participated considered CVD as their leading cause of death, and breast cancer was identified as the main health concern [4]. These data motivated the AHA to launch a national campaign to raise awareness and educate about the risks of CVD in women. In 2013, Mosca and colleagues published 15-year trends in awareness and knowledge about CVD in American women and reported that, while marked improvements had been made in overall awareness of CVD as the leading cause of death in women, there were significant disparities across races, with white women showing more substantial improvements than black or Hispanic women [5]. Unfortunately, African American and Hispanic population presented the highest cardiovascular (CV) risk factor prevalence in USA, resulting in greater CVD rates [6]. As a matter of fact, African-American women have higher rates of hypertension and lower rates of blood pressure control than white American women—factors which likely explain the elevated rates of stroke observed in them [7]. In addition, the prevalence of metabolic syndrome is very high in Hispanic women in the U.S, especially in Mexican-American women older than 50 years old [8,9].

A similar scenario is observed in Hispanic women who lived in Latin America. As a glance, in Chile there has been an increase in CV risk factor burden across the population over the past 20 years. In the CARMELA (Cardiovascular Risk Factor Multiple Evaluation in Latin America) study, Chilean women had the highest rates of smoking from the seven participating Latin American countries [10]. Furthermore, in the 2016 Chilean National Survey, the prevalence rates of obesity and diabetes in women were around 38% and 14%, respectively, being some of the highest prevalence in Latin American women [11]. As a consequence, approximately one of three women die of CVD in Chile [12], however, there are no published data about awareness, knowledge, and perceptions of CVD in them, with the exception of a 2010 survey conducted in mothers of students from a sampling of schools in Santiago [13]. This information is important to ascertain for public health since knowledge about CVD risk often encourages individuals to take preventive action.

The published data from the United States highlight the paucity reported information on the awareness and knowledge of CVD in Hispanic women in other countries. For these reasons, the purpose of this study was to assess awareness and knowledge about CVD and its risk factors in Chilean women.

## Methods

Cross-sectional study corresponding to the recruitment phase of the prospective ESCI project (Spanish acronym for ‘Study of ideal cardiovascular health in women’), which its main purpose was to assess the impact of an SMS-text message intervention compared to no intervention in promoting the seven ideal CV healthy metrics and behaviors as presented by AHA in 2010 [14]. ESCI sought to collect information from women between 35 to 70 years old, residents of Santiago de Chile, Metropolitan Region, which includes 52 cities or boroughs representing 40% of the entire Chilean population [15]. The study was reviewed and approved by the Santiago East Metropolitan Region Ethics Committee. All participants signed an informed consent.

A total target sample size of around 740 women was determined, considering a ~15% participant loss between the two baseline home visits. The sample design was probabilistic, multistage, and geographically stratified. Before sample selection, nine strata were formed by crossing three socioeconomic levels and three age groups at the city level. The three socioeconomic strata were formed by estimating the average of the total per capita income of the household residents of the 52 cities using data from the 2011 National Socioeconomic Characterization (CASEN) Survey [16]. The cities were grouped into three strata formed according to the predominance of women of specific age ranges (35 to 44, 45 to 54 and 55 to 70 years old) concerning the population distribution in the Metropolitan Region. In each of the nine sampling strata formed by the above design, two cities were selected as the primary sampling units (PSU), yielding 18 PSU (Supplementary Appendix, Table S1). From each PSU, 12 blocks were selected using a systematic random selection method with probability proportional to size. In each block a registration of the residential dwellings was carried out. Within the 18 cities, 217 blocks and 2717 housing units were selected. Screening interviews to identify eligible women were conducted in 1817 of these housing units. Finally, 1027 eligible women were selected (only one woman per household could participate in the study), and among those 723 were successfully recruited to participate. This recruitment was held between May and August 2015.

Eligible women were aged 30 to 75 years with access to cellular phones (in use and functioning, a requirement of ESCI). Women with a history of stroke and/or myocardial infarction and/or renal failure on dialysis, and pregnant women were excluded. The sample design and fieldwork were conducted by the Centro de Estudios y Encuestas Longitudinales of the Pontificia Universidad Católica de Chile.

### Data collection and survey design

ESCI baseline data were collected during two home visits of the selected women. The survey was completed during the first visit, and anthropometric measurements and blood and urine samples were collected during the second visit. The present study data have been obtained from the information recorded in the first visit. The survey takers were all female, and they conducted face-to-face interviews during which the survey was completed using an electronic tablet. Before the administration of the survey, all participants signed an informed consent agreeing to participate in the study.

The survey was carried out by a group of cardiologists, dietitians, nurses, and an expert in survey methodology. It was designed to be administered using an electronic tablet and included eleven questions about CHD and stroke awareness from the AHA survey on awareness of CVD in women (see Supplementary appendix) [5]. The questions about identifying the most significant health problem facing women and the leading cause of death were ‘one-size-fits-all,’ meaning the interviewer asked the participant to select the most important answer if more than one was provided, as the survey had a drop-down list of potential responses from which only could one be selected. Potential answers were not read to the participant, and the interviewer had to classify the spontaneous answer within the available choices. The question, ‘what are the major causes of cardiovascular diseases that you know’ was a multiple-response question that allowed the interviewer to record all the answers provided by the participant. The response options were not read, and the interviewer had to classify the spontaneous answer(s) using the available terms. The interviewers administered all the surveys during a face-to-face interview with each woman.

The survey also included the Global Physical Activity Questionnaire (GPAQ) for physical activity [17,18], the Patient Health Questionnaire-9 (PHQ-9) for depressive symptoms assessment [19], and questions about history of traditional CV risk factors (i.e., hypertension, smoking, diabetes, hypercholesterolemia, family history of cardiovascular disease), gynecologic history, socioeconomic data (i.e., total family income), and sociodemographic data (i.e., marital status, years of education, type of health insurance, and employment status), based on 2011 CASEN survey [16].

### Definitions of variables

The socioeconomic level was classified into three groups: low, medium low, and medium high, determined by the geographic area of the respondent’s residence, and based on total per capita income of the resident households of each city [15].

Education level was ascertained by asking for the years of formal education and the highest qualification received by the participant. It was divided into three categories: low (defined as a primary school or less; 0–8 years), middle (assigned as complete or incomplete secondary education; 9–12 years), and high (defined as complete or incomplete university or technical studies; >12 years).

Cardiovascular risk factors were collected through self-report. Smoking was defined as smoking at least one cigarette in the last month. Hypertension, hypercholesterolemia, and diabetes were all defined as having a medical diagnosis for the particular condition with or without pharmacotherapy. A family history of CVD was defined as a history of myocardial infarction or cardiac death in a first-degree female relative <65 years old and/or male relative <55 years old. Leisure-time sedentarism was defined by the GPAQ and classified as those individuals who engaged in <150 minutes of moderate-intensity or <75 minutes of vigorous-intensity aerobic physical activity per week. Depression was defined by the PHQ-9 score and classified as mild-to-moderate depressive symptoms (10 to 19 points) and major depression (≥20 points).

### Statistical analysis

Results are presented as mean ? standard deviation (SD) or percentage, as appropriate. ANOVA and Chi-square tests were considered. Prevalence rates and their comparison are based on weighted logistic regression models. All the reported data and statistical tests were adjusted according to the corresponding expansion factors in the sample design, representing the population of women in Santiago de Chile, Metropolitan Region. Therefore, the data reported in this study have application to the entire population of women aged 35 to 70 years who live in this region of the country. R version 4.1 was used for all analyses.

## Results

### Demographics and clinical characteristics

A total of 723 women participated in the study, with a mean age of 51 ??9 years. Approximately two-thirds of the participants (68.4%) were married or living together, the vast majority had children (96.0%), and 54.1% had a middle education level (Table 1). Of all participants, 36.5% were in the low socioeconomic level, and 79.6% had health insurance provided by the public health system.

**Table 1:** Demographics and clinical characteristics of study participants.

Characteristic	N = 723

**Age, years (mean ± SD)**	51 ± 9
**Age group, %**	
35–44 years	26.0
45–54 years	38.6
55–70 years	35.4
**Education level, %**	
Low (primary school or less; 0–8 years)	28.3
Middle (complete or incomplete secondary education; 9–12 years)	54.1
High (complete or incomplete university or technical studies; >12 years)	17.6
**Socioeconomic level, %**	
Low	36.5
Medium low	34.6
Medium high	28.9
**Marital status, %**	
Married or living together	68.4
Separated or divorced	14.3
Single	11.9
Widowed	5.0
**Number of children, %**	
none	4.0
1–2	45.1
>2	50.9
**Type of health insurance, %**	
Public	79.6
Private	17.4
Armed forces	2.1
Other/Don’t know	0.9
**Employment status, %**	
Employed (full- or part-time)	55.4
Student	0.7
Retired	2.7
Homemaker	41.1
**Self-reported personal medical history, %**	
Hypertension	37.8
Diabetes	15.6
High cholesterol	60.4
Current smoking	36.9
Overweight or Obesity	77.1
CVD family history	26.4
**Prevalence based on GPAQ and PHQ-9 questionnaires %**	
Leisure-time sedentarism	89.0
Depressive symptoms (mild to moderate)*	19.4
Depressive symptoms (severe)*	3.7

Data are mean ± SD except where indicated. * Depression was defined by the Patient Health Questionnaire-9 [ PHQ-9 ] score. Mild-to-moderate depressive symptoms was defined as a PHQ-9 score of 10 to 19 points and major depression as a score of ≥20 points. GPAQ, Global Physical Activity Questionnaire; PHQ-9, Patient Health Questionnaire-9; SD, standard deviation.

The majority of participants had ≥1 CV risk factor (98.9%), with 65.1% reporting ≥3. Leisure-time sedentarism was the most prevalent CV risk factor (89.0%), followed by overweight/obesity (77.1%). Within the 12 months before being surveyed, 72.2% of the women had had blood pressure measured, 49.0% had had a blood glucose measurement, and 54.6% had had a lipid measurement.

### Awareness and perception of the risk of heart disease in women

**Figure 1:**
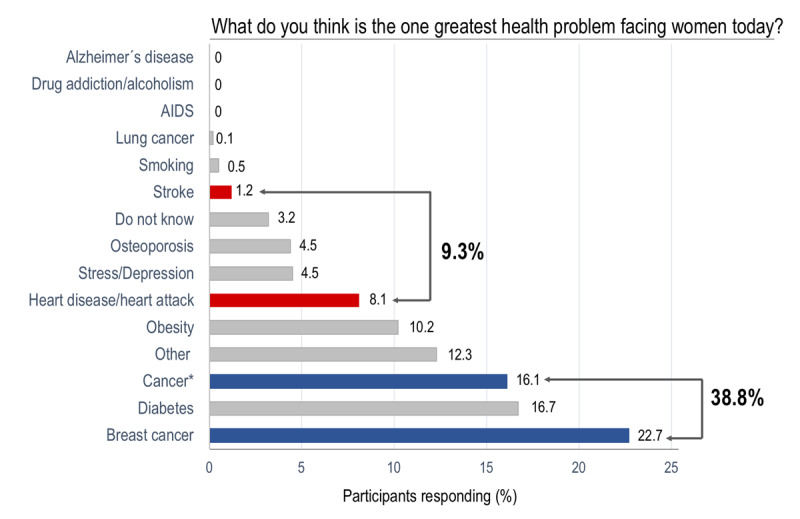
Distribution of participants’ responses about the main health problem for women. This question was a ‘one-size-fits-all,’ meaning the interviewer asked the participant to select the most important answer if she mentioned more than one. The survey had a drop-down list of potential answers, and only one could be selected. The potential answers were not read to the participant, and the interviewer had to classify the spontaneous answer within the available choices. * Cancer includes all malignancies except those in the breast and lung. AIDS, acquired immune deficiency syndrome.

When asked to identify the main health problem currently facing women, 22.7% of all participants said breast cancer and 16.1% responded cancer (general). In contrast, only 9.3 % identified CVD (heart disease/heart attack and stroke combined). Figure 1 shows the distribution of all responses to this question.

**Figure 2:**
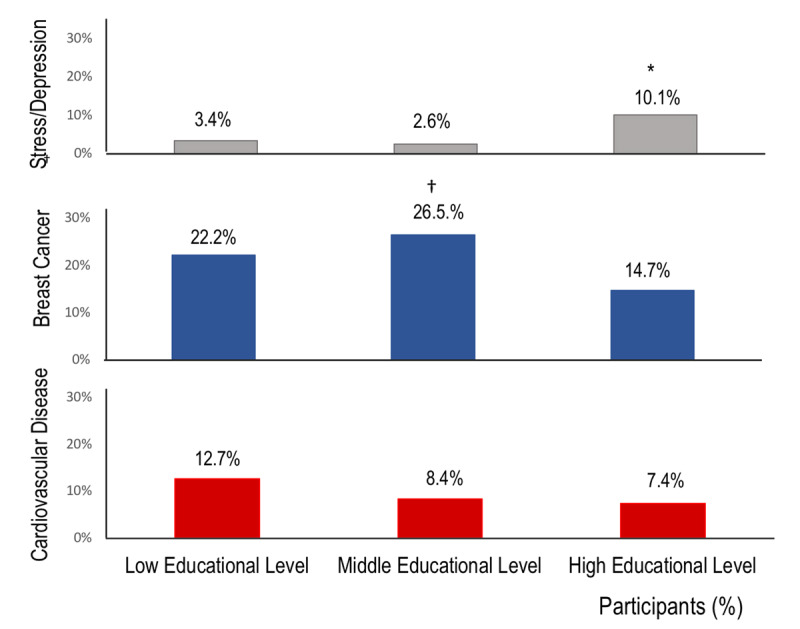
Perception of stress/depression, breast cancer, and cardiovascular disease as the main health problem for women, by education level. Participants were asked for the years of formal education and the highest qualification received. Three education levels were defined: low (primary school or less; 0–8 years), middle (complete or incomplete secondary education; 9–12 years), and high (complete or incomplete university or technical studies; >12 years). * p < 0.01 high vs middle and low education levels. † p < 0.01 middle vs high education level.

Responses by age and education level are reported in Table 2. Interestingly, more women from low (p = 0.08) and middle (p < 0.01) education level perceived breast cancer as the most significant health problem among women compared to their counterparts with high education level (Table S2, supplementary data). In contrast, women from high education level were more likely to identified obesity (p < 0.001) and mood disorders (stress and depression) (p < 0.01) as the leading health problem (Figure 2). No significant differences were observed by education level within general cancer and CVD (Table S3, supplementary data). No age and family CVD history-related differences were observed for this question.

**Table 2:** Awareness of greatest health problem facing women and leading cause of death among women by age and education level.

Response (%)	Age Group in years			p	Education level			p
	
	35–44 (n: 187)	45–54 (n: 279)	55–70 (n: 256)		Low (n: 205)	Middle (n: 391)	High (n:127)	

**Greatest Health Problem**								
Breast Cancer	18.6	26.7	21.6	NS	22.2	26.5	14.7	<0.01
Cancer (general)	18.7	19.4	14.1	NS	16.0	20.2	12.0	0.05
Heart disease/attack	9.1	8.5	10.2	NS	12.7	8.4	7.4	NS
Stroke	0.4	1.3	1.6	NS	0.7	1.2	1.5	NS
Diabetes	12.8	15.4	21.0	0.05	23.2	14.6	14.3	0.03
Obesity	13.7	11.0	7.2	0.07	3.1	10.5	18.1	<0.0001
Other	31.7	21.4	27.2	0.04	24.9	22.7	35.9	<0.01
Do not know	3.0	3.5	3.0	NS	5.4	2.8	1.4	0.10
**Leading Cause of Death**								

Breast Cancer	43.6	36.4	36.7	NS	36.7	38.1	41.2	NS
Cancer (general)	30.9	45.6	32.1	<0.001	39.1	40.3	26.5	<0.01
Heart disease/attack	9.7	9.4	24.2	<0.0001	16.4	11.7	20.2	0.03
Stroke	3.5	2.6	3.3	NS	2.3	2.3	5.7	NS
Diabetes	2.0	2.0	3.1	NS	2.5	2.2	2.8	NS
Obesity	1.0	0.2	0.8	NS	0.2	0.9	0.4	NS
Other	11.7	9.0	4.4	0.01	7.2	7.4	10.6	NS
Do not know	8.8	5.4	1.5	<0.001	2.4	5.4	6.7	NS

**Figure 3:**
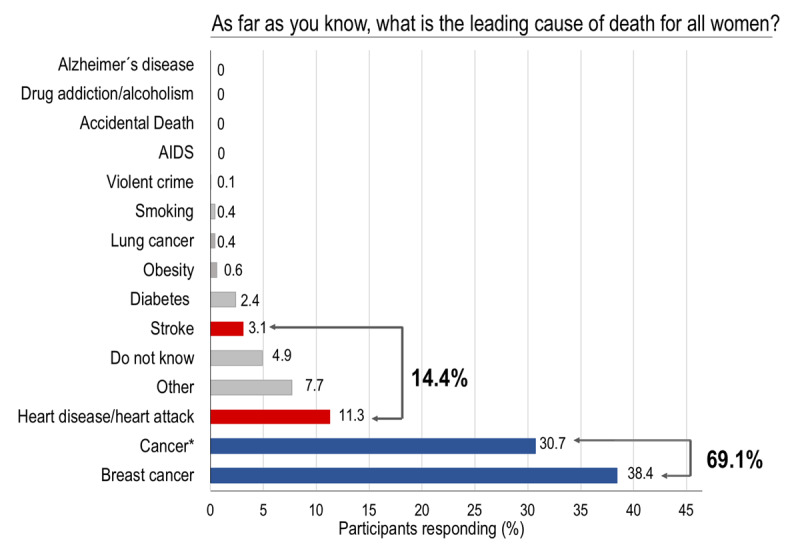
Distribution of participants’ responses about leading cause of death in women. This question was a ‘one-size-fits-all,’ meaning the interviewer asked the participant to select the most important answer if she mentioned more than one. The survey had a drop-down list of potential answers, and only one could be selected. The potential answers were not read to the participant, and the interviewer had to classify the spontaneous answer within the available choices. * Cancer includes all malignancies except those in the breast and lung. AIDS, acquired immune deficiency syndrome.

In response to the question, ‘what do you believe is the leading cause of death for women,’ 38.4% and 30.7% of the participants mentioned breast cancer and general cancer, respectively. Only 14.4% identified CVD (heart disease/heart attack and stroke combined) (Figure 3). No significant differences were observed by education level, except among middle-level women who have significantly less perception of CVD as the leading threat (p = 0.01) (Table S4, supplementary appendix). Besides, women ≥ 55 years old identified CVD as the main cause of death more frequently than women <55 years (OR 2.9, 95% CI = 1.8–4.5, p < 0.0001), adjusted for socioeconomic level, family income and employment status in a multivariable logistic regression model (Table 3). Women with a family history of CVD had significantly better awareness of CVD as the main killer in women (OR 1.7, 95% CI = 1.1–2.6, p 0.02) (Table S5, supplementary data).

**Table 3:** Awareness of cardiovascular disease as leading cause of death in women by socioeconomic level, income, employment status and age (≥ 55 years or younger).

	O.R.	95% C.I.		P^a^

		Lower	Upper	

**Socioeconomic level**^b^				
Low	1.00			
Middle	1.33	0.72	2.46	NS
High	1.28	0.71	2.28	NS
**Income**^c^				
Under $210.000	1.00			
$ 210.001–$ 290.000	1.91	0.78	4.70	NS
$ 290.001–$ 380.000	0.72	0.26	1.98	NS
$ 380.001–$ 470.000	2.49	0.93	6.66	0.07
$ 470.001–$ 580.000	3.35	1.15	9.75	0.03
$ 580.001–$ 700.000	1.87	0.52	6.68	NS
$ 700.001–$ 880.000	0.63	0.13	2.99	NS
$ 880.001–$1.170.000	1.17	0.34	4.03	NS
$1.170.001-$1.800.000	12.19	3.44	43.20	<0.001
NR	4.44	1.76	11.19	<0.01
**Employment**^d^				
Employed	1.00			
Unemployed	1.77	1.09	2.85	0.02
**Age**				
35–54 y	1.00			
55–70 y	2.92	1.85	4.59	<0.0001

^a^ Logistic regression. ^b^ Likelihood ratio test p NS. ^c^ Likelihood ratio test p < 0.0001. ^d^ Likelihood ratio test p < 0.01.

### Knowledge about cardiovascular disease and information sources

**Figure 4:**
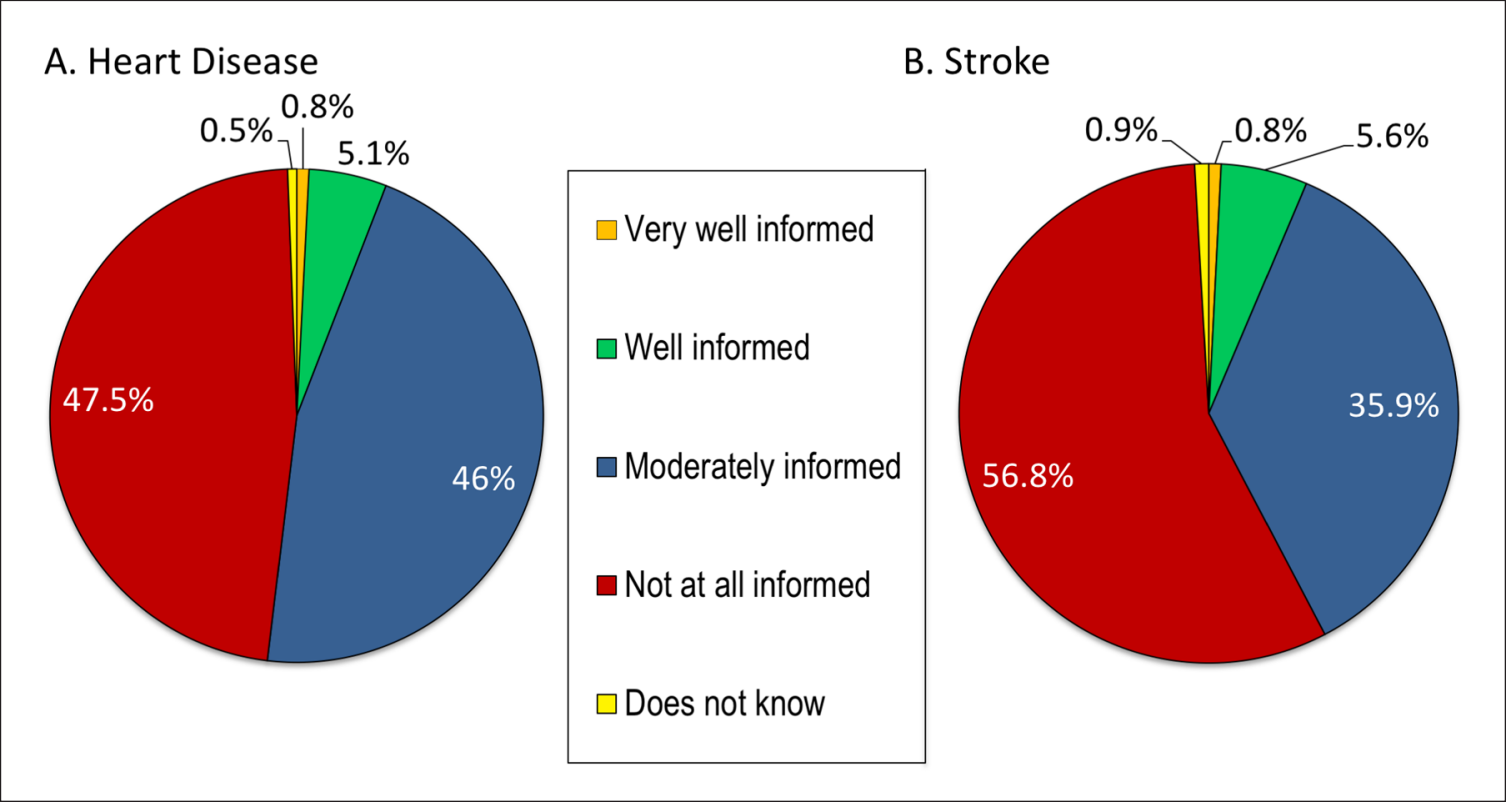
Distribution of participants’ responses about knowledge of heart disease **(A)** and stroke **(B)** in women.

When asked ‘how informed are you about CVD in women’ almost half the group responded ‘not at all informed,’ and only 6% of participants considered themselves to be well or very well informed (Figure 4A). No differences were observed by age or education level. The participants were less knowledgeable about stroke, with more than half responding that they were ‘not at all informed’ (Figure 4B). However, perceived knowledge of stroke differed significantly by education level, with women in the high education level more likely to respond that they were well informed than low education level women (p < 0.05).

Only 47% of participants indicated that they had seen, heard, or read information about heart disease within the 12 months before taking the survey. Of those women who responded positively, television was identified as the primary source for information (58%). Following TV, other sources of information included friends or relatives (27.2%), the Internet (7.3%), and healthcare professionals (6.7%). No differences by education level or age were observed for these responses except for the Internet: women in the high education level group identified the Internet as the main source of information significantly more frequently than women with middle or low education (p < 0.05).

### Knowledge about cardiovascular risk factors

When asked about to identify the primary causes of CVD, the main options selected were overweight (36%), smoking (32%), high cholesterol (30%), high blood pressure (22%), emotional stress (26%), lack of exercise (20%), diabetes (10%), and family history of CVD (8%). Overweight, sedentary lifestyle and family history of CVD were mentioned significantly more frequently by women with high education level (p < 0.05). Of the women who identified smoking as the main cause of CVD, 56% were active smokers. Similarly, 72.7% of women who stated that being overweight was a major CVD risk factor self-reported that they were overweight or obese, and 92.8% of women who listed physical inactivity as a CVD risk factor were classified as leisure-time sedentary by the GPAQ.

### Knowledge about symptoms of cardiac attack

When asked, ‘what signs do you associate with having a heart attack,’ responses were: chest pain (39%), pain or numbness that radiates to the back, neck, or arms (34.2%), shortness of breath (9%), tightness in the chest (7.2%), fatigue (5.2%), nausea (1.3%), and others (4.1%). Women with high education level identified fatigue and pain or numbness that radiates to the back, neck, or arms significantly more frequently than women with low or middle education level (p < 0.05 for both). In response to ‘what is the first thing you do when you suspect someone has a heart attack,’ 39% of the participants stated that they called emergency services.

## Discussion

The awareness of CVD as the leading cause of death in women from Santiago de Chile is deficient, as well as is the awareness of CVD as the main health problem. Prevalence of 14.4% and 9.3% of CVD as the leading cause of death and pivotal health problem in women are even worse as they were reported in AHA 1997 survey in the USA [4]. These results mandate great efforts by our national health authorities to focus on increasing awareness and knowledge about CVD in this population.

**Figure 5:**
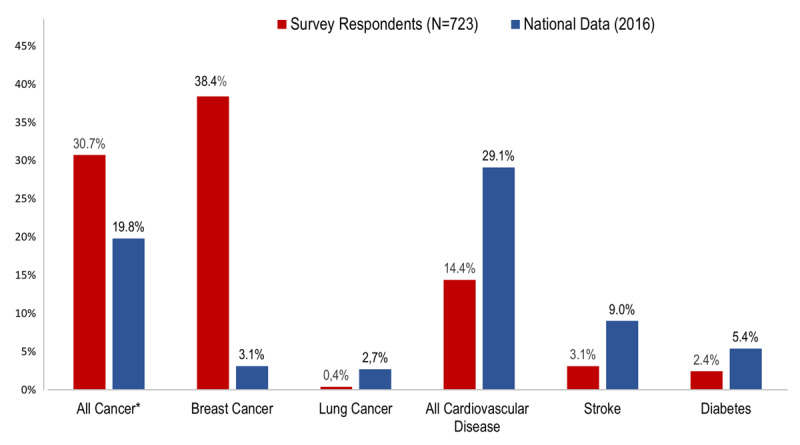
Perceived leading cause of death vs actual mortality data from national statistics in Chile (2016). Data from national statistics are derived from National Institute of Statistics (INE): mortality in women ≥35 years by all causes [12]. Survey respondents are the 723 women who participated in the current study. * All cancer includes all malignancies except those in the breast and lung.

CVD remains the leading cause of death in both men and women in Chile. Indeed, the latest statistical reports of deaths in Chile revealed that CVD-related mortality is higher in women than in men, accounting for 28.9% of the total deaths in women than 26.2% in men [12]. Ischemic heart disease and stroke are the principal causes of CVD-related deaths. Given these data, it would be expected that Chilean women would have some awareness and knowledge about the genuine threat CVD poses to them; however, our study suggests this is not the case. When compared with the latest statistics on causes of death in Chilean women, our results for awareness about the leading causes of death indicate an apparent discordance between reality and perception (Figure 5). A great effort must be made by all healthcare providers to bridge this gap. Besides, government authorities in our country need to include not only education about CVD risk factors but also information about the threat CVD poses in their agendas.

In our study, 69.1% of respondents considered cancer (combined breast and general cancer) as their leading killer, and only 14.4% identified CVD. This level of CVD awareness is about the same as that observed in African American women in the first survey in the US (15%), representing the worst awareness in US American women among different ethnicities [4]. However, after 15 years, a substantial improvement in CVD awareness has been observed across all the US women, including African American, with an increase from 15% to 36% [5]. By contrast, Latin American women are still unaware that CVD is the leading cause of death in them. As it has been demostrated in our study, as well as in two other surveys, one conducted in Uruguay and the other in Argentina [20,21]. In the study from Uruguay, 59% of the participants responded that cancer (both general and breast) was the leading cause of death compared with 12% who answered CVD [20]. The survey performed in Argentina reported similar findings with 54% and 20% for cancer (general and breast) and CVD, respectively [21]. Based on these data, Chilean women appear to have the most significant gap for awareness between CVD and cancer as the leading cause of death. Importantly in our study, women ≥55 years old were around 2.9 times more informed about CVD as their leading killer than their younger counterparts. This finding was somewhat anticipated, since the onset of CVD in women is later in life, around menopause, which for women represents a period of CV risk worsening. This information is consistent with other surveys, like the one recently published by Gooding et al., in which in American women aged between 15 to 24 years old, awareness of CVD was extremely low (10%) [22]. This finding emphasizes the need for education campaigns for women of all ages, including teens.

An interesting finding was the better awareness of CVD death in women with a family history of CVD. We found 1.7 times more perception than those without it, even higher than those reported by Mosca et al. in 2006 [23]. Probably, this relates to the genetic worry of having the same biological disease as their relatives [24]. Hamilton and Lobel reported that women with a family history of CVD perceived higher personal risk than women without a family history [25]. This finding can also be expected due to increased access to information and education on CVD within the family. Either way, the physician’s questions about genetic family history can improve awareness and allow behavioral changes [26].

The same underestimation of CVD’s significant death threat is observed about the biggest health problem facing women. Our study showed that participants did not consider CVD to be the leading health problem for women, and it ranked well below breast cancer and other cancers. Besides, it was interesting that approximately 5% of participants identified stress and/or depression as the leading health problem for women. This finding is not entirely unexpected, given the prevalence of depression in this cohort, similar to data reported in Chilean national surveys. The prevalence rates of permanent stress and depressive symptoms in women reported in the Chilean National Survey (2009–2010) in Santiago, Metropolitan Region were 12.1% and 25.7%, respectively [27]. Curiously in our study, stress and/or depression were frequently perceived as the leading health problem by women with high education level, whereas the National Survey showed a higher prevalence of depressive symptoms in individuals with low education level. From our perspective, the identification of depression and permanent stress as the leading health problem is compelling, since both conditions are associated with CVD and are considered CV risk factors [28]. In recent years, there is increased understanding that stress cardiomyopathy is a consequence of an abnormal response to a catecholamine surge secondary to an emotional trigger, which is more common in female patients [29,30]. If Chilean women have these psychological conditions, physicians must identify and address them early.

Despite the lack of awareness about CVD as the main health problem for women, the respondents quickly recognized the mayor modifiable CV risk factors. This information is heartening, considering that CVD is partially preventable with risk factor control as early as possible. However, it was disappointing to find that the majority of the study participants who identified smoking, overweight/obesity, and physical inactivity as risk factors for CVD were themselves smokers, overweight/obese, or sedentary. Other authors have reported this discordance between knowledge and behaviors. Koniak-Griffin and colleagues described that CVD knowledge is not related to dietary habit score, body mass index, or physical activity in overweight, immigrant Latinas in Los Angeles, California [31]. Another study reaffirms this finding by showing that CV risk factor knowledge in women did not correlate with heart health behaviors [32]. To improve self-care and behavioral change, we must search for new strategies. Social stigmas or psychologic barriers make behavioral changes challenging to achieve, as shown in recent data from the Women’s Heart Alliance. In that study, the authors reported that 45% of the women canceled or postponed physician appointments until losing some weight; others mentioned difficulties finding time to exercise (41%) even though, their physicians had recommended them to do so [33]. This inability to modify unhealthy CV patterns is a significant barrier and is a call-to-action to identify tools that can achieve behavioral changes in the female population.

The findings from our study concerning sources of information for CVD are critically important. Television is the leading communication tool from which Chilean women receive information about current events and topics. Similar findings have been reported for women in other Latin American countries [20,21]. Therefore, any educational campaign in Chile should include a television component to achieve a real impact. Internet and social media approaches should be considered since their reach are growing in our region, and younger people could be more interested and involved in these topics. It would be desirable that social media communicators work together with health care professionals to communicate better the real threat CVD poses in the population.

### Limitations and strengths

This study has some limitations. It only included women from Santiago, Metropolitan Region. Therefore, the results cannot be extrapolated to the rest of the country. Our survey used selected questions from the AHA awareness survey (not the entire questionnaire). Thus, we lack information about how women communicate with their healthcare providers about heart disease and barriers to achieving a healthy heart lifestyle. Lastly, analyzing CV risk factors through self-report may have under- or overestimated the real prevalence rates of these conditions in the study sample.

The main strength of this study is that it is the first study of CVD awareness in women in Chile held in a large and epidemiologically representative sample of women from Santiago (Metropolitan Region), the most populous region in Chile. This study adds to the body of knowledge about these topics in Hispanic women from Latin America. Finally, our survey was administered during a face-to-face interview, which provides more reliable information than telephone surveys. People in telephone interviews tend to present themselves in socially desirable ways [34].

## Conclusions

In conclusion, women from Santiago, Chile, have a low awareness of CVD as the leading cause of death. Our results are similar to the data on African American and Hispanic women reported in the first survey commissioned by AHA about CVD awareness in 1997. These results demonstrate the urgent need for awareness campaigns about the impact of CVD in women by our public health authorities and medical societies to reduce CVD morbidity and mortality in Chilean women.

## Additional File

The additional file for this article can be found as follows:

10.5334/gh.534.s1 Supplementary Appendix. Methodology information and results (logistic regression analysis).

## Author Information

Paola Varleta and Mónica Acevedo were co-principal investigators of ESCI project.
