# Supplementary material for: Low Cardiovascular Disease Awareness in Chilean Women: Insights from the ESCI Project
Source: Glob Heart. 2020 Aug 12;15(1):55. doi: 10.5334/gh.534 (PMC7427666; doi:10.5334/gh.534)
Supplement: Supplementary Appendix. — Methodology information and results (logistic regression analysis). [file gh-15-1-534-s1.pdf]

Supplementary Appendix

I- Methodology

Table S1: Design of the sample units 2

Survey: Awareness questions (Spanish) 3

II- Results and Statistical analysis data

Table S2: Multivariate analysis of awareness of breast cancer and depression/stress as the greatest health problem 10

Table S3: Awareness Greatest Health Problem By Educational Level 11

Table S4: Multivariate analysis of awareness of CVD and breast cancer as main cause of death by educational level 12

Table S5: Multivariate analysis of awareness of CVD as main cause of death by CVD family history 13

Table S1 - Conformation of the nine boroughs/communes strata of Santiago, Metropolitan Region, and 18 primary sampling units according to socioeconomic level and age bracket for the study design

Boroughs by average household per capita income	Boroughs according to age group based on % of predominant age group of women at the Metropolitan Region			Total Boroughs or communes
	Greater % Women 35-44 years old	Greater % Women 45-54 years old	Greater % Women 55-70 years old	
**Lower Level from $141.355 to $173.879 pesos**	Renca<br>Lampa<br>Tiltil<br>San Joaquín*<br>Pudahuel*	Lo Espejo*<br>Alhué<br>Isla de Maipo*<br>Curacaví	San Pedro<br>San Bernardo<br>El Bosque<br>Recoleta<br>Cerro Navia*<br>La Pintana<br>María Pinto<br>Melipilla*<br>Lo Prado	18
**Medium Level from $176.645 to $223.884 pesos**	Puente Alto*<br>Colina*<br>San Ramón<br>Cerrillos<br>Buin<br>El Monte	Pedro Aguirre Cerda<br>Pirque<br>Estación Central*<br>Quilicura*<br>Macul<br>Padre Hurtado	La Granja*<br>Paine<br>La Cisterna<br>Conchalí<br>Quinta Normal*	17
**High Level from $234.950 to $1.412.161 pesos**	Huechuraba*<br>Peñaflor<br>Peñalolen*<br>Talagante<br>La Reina<br>Lo Barnechea<br>Providencia	San Miguel<br>San José de Maipo<br>Maipú*<br>La Florida*<br>Calera de Tango<br>Ñuñoa	Independencia<br>Santiago*<br>Las Condes*<br>Vitacura	17
**Total boroughs or communes**	18	16	18	52

Source: Own preparation based on the CASEN 2011 Survey[17] /
(*): Selected boroughs or communes

II- Cardiovascular disease and stroke awareness survey (In Spanish as was applied) . It only includes the questions related to awareness of CVD, therefore it doesn´t include the entire survey for ESCI project.

DTA.100 En general, ¿Cómo calificaría hoy su estado de salud?

LEER ALTERNATIVAS.

[ ] 1. Muy mala.

[ ] 2. Mala.

[ ] 3. Más o menos.

[ ] 4. Buena.

[ ] 5. Muy buena.

[ ] 8*. NO SABE.*

[ ] 9*. NO RESPONDE.*

AWA.010 ¿Cuál cree Ud. que es el mayor problema de salud que enfrenta la mujer actualmente?

NO LEER ALTERNATIVAS.

[ ] 1. Accidente cerebrovascular.

[ ] 2. Adicción a drogas/alcoholismo.

[ ] 3. Alzheimer.

[ ] 4. Cáncer de mama.

[ ] 5. Cáncer pulmonar.

[ ] 6. Cáncer (en general).

[ ] 7. Diabetes.

[ ] 8. Enfermedades cardiacas/ataque cardíaco.

[ ] 9. Obesidad.

[ ] 10. Osteoporosis.

[ ] 11. SIDA.

[ ] 12. Tabaco.

[ ] 13. Otro. Especifique: ______________________________________

[ ] 88*. NO SABE.*

[ ] 99*. NO RESPONDE.*

AWA.020 Hasta donde Ud. sabe, ¿Cuál es la principal causa de muerte para todas las mujeres?

NO LEER ALTERNATIVAS.

[ ] 1. Accidente cerebrovascular.

[ ] 2. Adicción a drogas/alcoholismo.

[ ] 3. Alzheimer.

[ ] 4. Cáncer de mama.

[ ] 5. Cáncer pulmonar.

[ ] 6. Cáncer (en general).

[ ] 7. Crimen violento.

[ ] 8. Diabetes.

[ ] 9. Enfermedades cardiacas/ataque cardíaco.

[ ] 10. Muerte accidental.

[ ] 11. Osteoporosis.

[ ] 12. SIDA.

[ ] 13. Tabaco.

[ ] 14. Otro. Especifique: ______________________________________

[ ] 88*. NO SABE.*

[ ] 99*. NO RESPONDE.*

AWA.030 ¿Ud. ha visto, escuchado o leído información acerca de enfermedades cardíacas dentro de los últimos 12 meses?

[ ] 1. Sí.

[ ] 2. No. *Pase a AWA.040*

[ ] 8*. NO SABE. Pase a AWA.040*

[ ] 9*. NO RESPONDE. Pase a AWA.040*

AWA.035 ¿Dónde vio, escuchó o leyó esta información?

NO LEER ALTERNATIVAS.

MARQUE TODAS LAS QUE CORRESPONDA.

Permitir respuesta múltiple.

[ ] 1. En una revista.

[ ] 2. En la radio.

[ ] 3. En un libro.

[ ] 4. En televisión.

[ ] 5. Información de un folleto, panfleto, etc.

[ ] 6. Entregada por el médico, enfermera u otro profesional de la salud.

[ ] 7. En un periódico.

[ ] 8. En internet.

[ ] 9. De un amigo o pariente.

[ ] 10. Biblioteca.

[ ] 11. Otro. Especifique: ______________________________________

[ ] 88*. NO SABE.*

[ ] 99*. NO RESPONDE*

AWA.040 ¿Ud. ha visto, escuchado (oído) o leído algo acerca del símbolo “vestido rojo” o “red dress symbol”? MOSTRAR TARJETA AWA.040.

[ ] 1. Sí.

[ ] 2. No.

[ ] 8*. NO SABE.*

[ ] 9*. NO RESPONDE.*

AWA.050 ¿Algún médico, o personal médico como enfermera o nutricionista, le ha hablado de las enfermedades cardíacas o cardiovasculares?

[ ] 1. Sí.

[ ] 2. No.

[ ] 8*. NO SABE.*

[ ] 9*. NO RESPONDE.*

AWA.060 ¿Que tan informada está Ud. respecto a enfermedades cardíacas o cardiovasculares en la mujer? Ud. diría que está…

LEER ALTERNATIVAS.

[ ] 1. Muy bien informada.

[ ] 2. Bien informada.

[ ] 3. Medianamente informada.

[ ] 4. No informada en lo absoluto.

[ ] 8. *NO SABE.*

[ ] 9. *NO RESPONDE.*

AWA.070 ¿Qué tan informada está Ud. respecto al ataque cerebral o cerebrovascular en las mujeres? Ud. diría que está...

LEER ALTERNATIVAS.

[ ] 1. Muy bien informada.

[ ] 2. Bien informada.

[ ] 3. Medianamente informada.

[ ] 4. No informada en lo absoluto.

[ ] 8. *NO SABE.*

[ ] 9. *NO RESPONDE.*

AWA.080 Basándose en lo que Ud. sabe, ¿Cuáles son las mayores causas de enfermedades cardíacas o cardiovasculares?

NO LEER LAS ALTERNATIVAS.

MARQUE TODAS LAS QUE CORRESPONDA.

Permitir respuesta múltiple.

[ ] 1. Historia familiar de enfermedades cardíacas (cardiovasculares).

[ ] 2. Envejecimiento.

[ ] 3. Estar con sobrepeso.

[ ] 4. Diabetes.

[ ] 5. Beber alcohol.

[ ] 6. Presión arterial alta (hipertensión).

[ ] 7. Colesterol alto.

[ ] 8. Triglicéridos altos.

[ ] 9. Bajos niveles de estrógenos.

[ ] 10. Menopausia.

[ ] 11. No hacer ejercicio.

[ ] 12. Fumar.

[ ] 13. Estrés.

[ ] 14. Accidente cerebrovascular.

[ ] 15. Otra. Especifique: ____________________________________

[ ] 88*. NO SABE.*

[ ] 99*. NO RESPONDE.*

AWA.090 Basado en lo que Ud. sabe ¿Qué signos de alarma asocia Ud. con tener un ataque cardíaco?

NO LEER ALTERNATIVAS.

MARQUE TODAS LAS QUE CORRESPONDA.

Permitir respuesta múltiple

[ ] 1. Dificultad respiratoria.

[ ] 2. Dolor de pecho.

[ ] 3. Dolor o adormecimiento que se irradia a espaldas, cuello o brazos.

[ ] 4. Fatiga.

[ ] 5. Nauseas.

[ ] 6. Opresión (presión) en el pecho.

[ ] 7. Otra. Especifique: __________________________________

[ ] 88*. NO SABE.*

[ ] 99*. NO RESPONDE.*

AWA.100 Si Ud. cree que alguien está teniendo un ataque cardíaco, ¿Qué es la primera cosa que Ud. haría?

NO LEER ALTERNATIVAS.

[ ] 1. Llevarlo a un hospital/clínica.

[ ] 2. Llevarlo a ver al doctor.

[ ] 3. Llamar a la ambulancia de urgencia (131, SAMU).

[ ] 4. Llamar a su pareja o un familiar.

[ ] 5. Otra. Especifique: ____________________________________

[ ] 88*. NO SABE.*

[ ] 99*. NO RESPONDE.*

Table S2. Multivariate analysis of awareness of **breast cancer and depression**/stress as the greatest health problem in women by educational level

	Model 1			Model 2		
	**Breast Cancer**					
Educational Level[a]	OR		P[b]	OR		P[b]
High	Ref 1.0			Ref 1.0		
Middle	2.08	[1.28 -3.40]	< 0.01	2.05	[1.25 -3.36]	< 0.01
Low	1.65	[0.95 -2.87]	0.08	1.63	[0.95 -2.85]	0.08
	**Depression/stress**					
Educational Level[a]	OR		P[b]	OR		P[b]
High	Ref 1.0			Ref 1.0		
Middle	0.23	[0.09 -0.60]	< 0.01	0.25	[0.09 -0.65]	< 0.01
Low	0.31	[0.16 -0.62]	< 0.001	0.33	[0.16 -0.67]	< 0.01

[a] Logistic regression
[b] Likelihood ratio test p < 0.01
Model 1: Crude OR (univariate)
Model2: Adjusted OR with age.

Table S3. Awareness of Greatest Health Problem By Educational Level

Adjusted by Age

	**Educational level**			
Greatest health problem	Lower	Middle	High	P[a]
Stroke %	0.7	1.2	1.5	NS
Breast Cancer %	22.2	26.5	14.7	< 0.01
Lung Cancer %	0.0	0.1	0.0	NS
Cancer (in general) %	16.0	18.0	12.0	NS
Diabetes %	23.2	14.6	14.3	0.03
Cardiovascular Disease %	5.1	3.6	1.9	NS
Obesity %	2.3	10.5	18.1	< 0.0001
Smoking (%	0.0	0.7	0.6	NS
Other %	18.8	17.5	32.7	< 0.001
Do not know %	5.4	2.8	1.4	0.10

[a] Logistic regression, adjusted by age.

Table S4. Multivariate analysis of awareness of health conditions as main cause of death in women by educational level

	Model 1			Model 2		
	**Breast Cancer**					
Educational Level[a]	OR	95% C.I.	P[b]	OR	95% C.I.	P[b]
High	Ref 1.0			Ref 1.0		
Middle	0.88	[0.61 -1.28]	NS	0.87	[0.56 -1.35]	NS
Low	0.83	[0.54 -1.28]	NS	0.92	[0.63 -1.35]	NS
	**CVD**					
Educational Level[a]	OR	95% C.I.	P[b]	OR	95% C.I.	P[b]
High	Ref 1.0			Ref 1.0		
Middle	0.52	[0.32 -0.86]	0.01	0.41	[0.24 -0.69]	< 0.01
Low	0.78	[0.45 -1.34]	NS	0.60	[0.34 -1.06]	NS

[a] Logistic regression
[b] Likelihood ratio test $p < 0.01$
Model 1: Crude OR (univariate)
Model2: Adjusted OR with age.

Table S5. Multivariate analysis of awareness of CVD as main cause of death in women by CVD family history

	Model 1			Model 2		
	**CVD**					
Family History of CVD[a]	OR	95% C.I.	P[a]	OR	95% C.I.	P[b]
Absent	Ref 1.0			Ref 1.0		
Present	1.72	[1.12 -2.66]	0.01	1.72	[1.11 -2.66]	0.02

[a] Logistic regression
[b] Likelihood ratio test p = 0.03
Model 1: Crude OR (univariate)
Model 2: Adjusted OR with educational level.
